# Molecular phylogenetic analysis of *Echinococcus multilocularis* from horses raised in Canada or Japan, using mitochondrial cytochrome *b* gene–targeted PCR

**DOI:** 10.1016/j.fawpar.2024.e00219

**Published:** 2024-01-13

**Authors:** Tatsuro Hifumi, Tetsuya Tanaka, Ichinosuke Suzu, Miho Sato, Kohei Akioka, Chiaki Fujimata, Ryohei Shinkai, Tsutomu Maeda, Kodai Kusakisako, Hiromi Ikadai, Noriaki Miyoshi

**Affiliations:** a Laboratory of Veterinary Histopathology, Joint Faculty of Veterinary Medicine, Kagoshima University, 1-21-24 Korimoto, Kagoshima 890-0065, Japan; b Laboratory of Infectious Diseases, Joint Faculty of Veterinary Medicine, Kagoshima University, 1-21-24 Korimoto, Kagoshima 890-0065, Japan; c Kumamoto Prefectural Meat Inspection Office, 1314 Sosaki, Shichijo-machi, Kikuchi, Kumamoto 861-1344, Japan; d Fukuoka Prefecture Meat Safety Inspection Center, 4-5-34 Futsukaichichuo, Chikushino, Fukuoka 818-0072, Japan; e Laboratory of Veterinary Parasitology, School of Veterinary Medicine, Kitasato University, 23-35-1 Higashi, Towada, Aomori 034-8628, Japan

**Keywords:** Alveolar echinococcosis, Horse, Meat inspection, Mitochondrial cytochrome *b*, Cob-gene

## Abstract

Alveolar echinococcosis is a zoonotic disease caused by a larval-stage *Echinococcus multilocularis* infection. Geographical haplotyping targeting the parasite's mitochondrial cytochrome *b* (*cob*) gene has been reported for isolates from definitive and intermediate hosts (wild canids and rodents); however, there are limited reports on strain typing for the dead-end host, the horse, which could act as a sentinel for E*. multilocularis*. Accordingly, we investigated the diversity of *E. multilocularis* in isolates obtained from slaughtered Japanese and Canadian horses originating from the Iburi and Hidaka regions in Hokkaido and from Alberta, respectively, with PCR and haplogroup analyses targeting *cob* gene sequences obtained. Seventy horses were diagnosed with alveolar echinococcosis based on histopathology and *cob*-gene PCR testing. The *E. multilocularis* detected in these horses was classified as either an Asian (for Hokkaido-raised horses) or a European (for Alberta-raised horses) haplogroup, based on the obtained *cob*-gene sequence analysis. In addition, haplotype network analysis revealed that *E. multilocularis* isolated from Hokkaido-raised horses is highly homologous to Kazakhstan isolates, and *E. multilocularis* isolated from Alberta-raised horses is highly homologous to Austrian isolates. The results of this study suggest that *cob*-gene-targeted PCR analysis could be useful for the geographical genetic characterization of *E. multilocularis* isolated from horses.

## Highlights

• Molecular characterization of *Echinococcus multilocularis* from Japanese and Canadian horses based on *cob*-gene.
• Epidemiological status of *E. multilocularis* in the regions of Japan and Canada where horses were raised for slaughter.
• Effectiveness of PCR targeting *cob* gene for geographical characterization of *E. multilocularis* from horses.

## Graphical abstract

**Figure:**
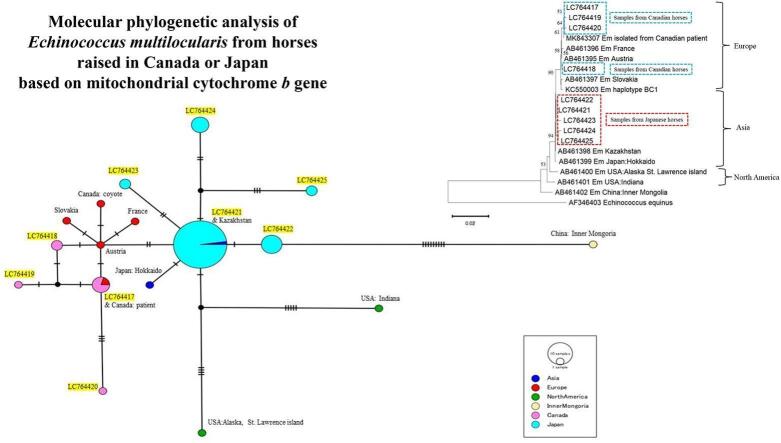

## 1 Introduction

Alveolar echinococcosis, a zoonotic disease with the potential to pose a significant threat to public health, is caused by the larval stage of *Echinococcus multilocularis* (Deplazes and Eckert, 2001). This parasite is endemic to many areas across North America, Europe, and Asia (Eckert and Deplazes, 2004). The life cycle of *E. multilocularis* involves wild canids and dogs as definitive hosts, and small rodents as intermediate hosts (Deplazes and Eckert, 2001; Eckert and Deplazes, 2004). *E. multilocularis* can also reportedly infect various dead-end host species, including pigs (Knapp et al., 2021; Karamon et al., 2023), and horses (Tomczuk et al., 2020), which are regarded as potential sentinel animals for this parasite.

Horses become infected through the oral ingestion of eggs from definitive host feces, and lesions caused by larval *E. multilocularis* infection are commonly found in their livers (Goto et al., 2010). Alveolar echinococcosis has been reported in slaughtered horses in Japan (Goto et al., 2010; Ueno et al., 2012) and Poland (Tomczuk et al., 2020). Previously, we investigated cases of grayish-white hepatic solid nodules found in horses slaughtered in Japan and demonstrated larval *E. multilocularis* infections suspected to have originated in Japan (Hifumi et al., 2015) or Canada (Hifumi et al., 2021).

In previous reports, alveolar echinococcosis was mainly identified in slaughtered horses, with a definitive diagnosis based on the results of histopathological examination and polymerase chain reaction (PCR) testing (Goto et al., 2010; Hifumi et al., 2015). PCR testing targeting mitochondrial DNA can determine the characteristic geographical patterns of various parasitic diseases (Khan et al., 2020; Nguyen et al., 2020). One potential target gene, mitochondrial cytochrome *b* (*cob*) encodes a protein located in the mitochondria of eukaryotic cells that operates within the electron transport chain (Esposti et al., 1993). Differing *cob* gene sequences can be exploited in phylogenetic analysis for a range of organisms (Farias et al., 2001; Luyo-Acero et al., 2004). Taking advantage of this property, conventional PCR targeting the *cob* gene of E. multilocularis can be applied to classifying haplogroups as European, Asian, or North American (Nakao et al., 2009). For *E. multilocularis*, there are a number of reports on genetic examinations of isolates from the definitive and intermediate hosts, and these reports have included strain typing (Nakao et al., 2009; Gesy and Jenkins, 2015; Wu et al., 2017). However, the only previous investigation on strain typing in equine hosts was our earlier report on horses imported into Japan from Canada (Hifumi et al., 2021). Therefore, this study aimed to investigate the haplogroups of *E. multilocularis* obtained from horses raised in Japan and Canada based on the obtained *cob* sequences.

## 2 Materials and methods

### 2.1 Field samples

We targeted 187 hepatic nodules found during meat inspections at slaughterhouses in Japan between December 2020 and November 2022. This study included three slaughterhouses in Japan, which were in Kumamoto and Fukuoka Prefectures in southwestern Japan, and in Aomori Prefecture, which is in northern Japan. During the study period, the Kumamoto slaughterhouse handled 4483 horses, divided between horses of Japanese origin (*n* = 2236) and horses of Canadian origin (*n* = 2247), and the Fukuoka and Aomori slaughterhouses handled 1643 horses and 174 horses, respectively, all of which were horses of Japanese origin.

**Fig. 1:**
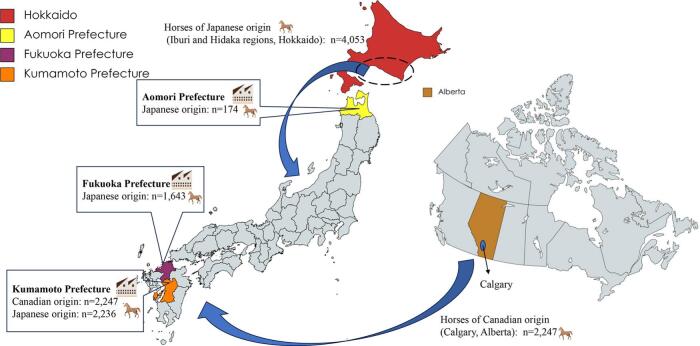
Horses slaughtered in Japan (December 2020–November 2022): Origins and slaughterhouse locations. Map of Japan showing the four provinces included in this study: Hokkaido, Aomori Prefecture, Fukuoka Prefecture, and Kumamoto Prefecture. The nodules used in this study were collected from the slaughterhouses in Aomori Prefecture, Fukuoka Prefecture, and Kumamoto Prefecture. Most horses in Japan were born in the Iburi and Hidaka regions of Hokkaido, where alveolar echinococcosis is endemic. Additionally, there is a map of Canada showing Calgary, the province of Alberta that was included in this study. Before being imported to Japan, the horses originating in Canada were born and raised in Calgary, Alberta, where alveolar echinococcosis is endemic. This map was prepared using MapChart (https://www.mapchart.net/).

The nodules evaluated in this study were obtained from 64 horses slaughtered in Kumamoto Prefecture, 120 horses slaughtered in Fukuoka Prefecture, and three horses slaughtered in Aomori Prefecture. Seventeen of 64 horses slaughtered in Kumamoto Prefecture had been imported from Canada and had undergone fattening in Kumamoto Prefecture for at least six months; these horses had been born and raised in the Canadian province of Alberta. The other 47 horses slaughtered in Kumamoto Prefecture and all horses slaughtered in Fukuoka or Aomori Prefecture were of Japanese origin and had been born and then raised for at least one year on Japan's northern island of Hokkaido (Iburi and Hidaka regions). None of the horses had shown adverse clinical signs prior to slaughter. The origins of slaughtered horses and slaughterhouse locations are shown in Fig. 1.

One nodule was collected from each horse that had no clinical signs before slaughter. When multiple hepatic nodules were found, only the largest nodule was collected. Each collected nodule was bisected; one part was submitted for histopathological examination, and the other part was stored frozen at −20 °C for genetic examination.

### 2.2 Histopathological examination

Nodules were fixed in 10% neutral-buffered formalin, decalcified with 10% formic acid solution for three days, and neutralized with 5% sodium sulfate solution for sixteen hours. Then they were routinely embedded in paraffin, sectioned in 3 μm, and stained with hematoxylin and eosin (HE) and periodic acid-Schiff (PAS).

### 2.3 DNA extraction, *cob-*gene-targeted PCR testing, and molecular phylogenetic analysis

A 25 mg tissue sample was collected from each nodular lesion, and the DNA was extracted using a DNeasy Blood & Tissue Kit (Qiagen, Hilden, Germany) in accordance with the manufacturer's instructions. PCR testing was performed to amplify a 694 bp fragment of the *cob* gene of *E. multilocularis*, as previously reported (Gesy and Jenkins, 2015). The primers and conditions were as follows: forward (5’-TGCGTTATTGGCATATGGTAG-3′) and reverse (5′- GTGCCACCCTCAGTTGGTACT-3′) primers were applied, with initial denaturing at 95 °C for 3 min, followed by 40 cycles at 94 °C for 30 s, 54 °C for 30 s, and 72 °C for 45 s, and a final extension at 72 °C for 5 min. The locations and sequences corresponding to the above-mentioned primers are shown in Supplementary Fig. 1. An amplification reaction was performed in a 12.5 μl (total volume) mixture, containing 1.0 μl of the DNA template, 6.25 μl of a 2× Gflex PCR buffer (Takara Bio Inc., Shiga, Japan), 0.15 μl of each primer (10 μM), 0.15 μl of Tks Gflex DNA polymerase (1.25 U/μl; Takara Bio Inc.), and 4.8 μl of sterile distilled water. The PCR-amplified products were analyzed by electrophoresis on 1.5% agarose gel. The PCR-positive bands were excised from the 1.5% agarose gel and purified with NucleoSpin® Gel and PCR Clean-up (Takara Bio Inc., Shiga, Japan). The PCR-amplified products from positive samples were directly sequenced using a 3730xl DNA Analyzer (Thermo Fisher Scientific, Waltham, Massachusetts, USA). The obtained sequences were compared with sequences in the National Center for Biotechnology Information's nucleotide database (https://blast.ncbi.nlm.nih.gov/Blast.cgi).

Based on the obtained sequences, molecular phylogenetic tree analysis with the neighbor-joining method based on Tamura-Nei model was performed using MEGA 11 software (Tamura et al., 2021). The sequences were compared to previously published *cob* sequences of *E. multilocularis* (Nakao et al., 2009; Gesy and Jenkins, 2015; Massolo et al., 2019). Additionally, haplotype network analysis was performed using the TCS Network in PopART version 1.7 software (Leigh and Bryant, 2015).

## 3 Results

### 3.1 Alveolar echinococcosis diagnosis and regional prevalences

According to our previous report (Hifumi et al., 2021), cases that had laminated layers histopathologically and tested positive for *cob* PCR testing or cases that did not have laminated layers histopathologically and tested positive for *cob* PCR testing were diagnosed with alveolar echinococcosis.

Histopathologically, the nodules collected from all 187 horses were fibrous nodules consisting of mature collagen fibers, accompanied by central necrosis and mild-to-moderate infiltration of eosinophils, lymphocytes, and macrophages at the periphery of the nodule. Sixteen of the 187 horses had characteristic laminated layers of *E. multilocularis* in the lesions and tested positive with *cob* PCR testing. In addition, 54 horses without the characteristic laminated layers of *E. multilocularis* in the lesions histopathologically tested positive with *cob* PCR testing. Representative photographs of the histopathology and *cob* PCR testing are shown in Supplementary Figs. 2 and 3. A total of 70 horses were diagnosed with alveolar echinococcosis. These horses originated in Japan (62 horses) and Canada (8 horses). Based on these figures and the total numbers of slaughtered horses, the equine alveolar echinococcosis prevalence rates in this study were 1.53% for the Hokkaido-raised horses (62/4053 horses), and 0.36% for the Alberta-raised horses (8/2247 horses), respectively. Data including origin, breed, sex, age, nodule size, histopathology, *cob* PCR, GenBank accession number, and haplogroup for 70 horses diagnosed with alveolar echinococcosis are shown in Supplementary Table 1.

### 3.2 DNA sequencing and molecular phylogenetic analysis

The obtained *cob* sequences have been registered in the database of the DNA Data Bank of Japan (https://www.ddbj.nig.ac.jp/index-e.html; GenBank accession numbers LC764417, LC764418, LC764419, LC764420, LC764421, LC764422, LC764423, LC764424, and LC764425).

The sequences isolated from the 62 Japanese horses (LC764421-764425) showed 99.43% to 100% homology with those for *E. multilocularis* isolated in Kazakhstan (GenBank accession number AB461398) and 99.28% to 99.86% homology with *E. multilocularis* isolated in Japan (GenBank accession number AB461399), which both belong to Asian haplogroups. When compared with the Kazakhstan isolate (GenBank accession number AB461398), LC764421 matched perfectly, LC764422 had one nucleotide substitution, LC764423 had two nucleotide substitutions, LC764424 had two nucleotide substitutions and one nucleotide insertion, and LC764425 had four nucleotide insertions. When compared with the Kazakhstan isolate, these five isolates had 98.95% to 100% homology in the amino acid sequences of the open reading frame region. When compared with the Kazakhstan isolate, LC764423 showed two amino acid mutations in the open reading frame. These five nucleotide sequences and amino acid sequences are shown in Supplementary Table 2.

The sequences we identified for isolates from eight Canadian horses (LC764417–764420) showed 99.43% to 99.86% homology with *E. multilocularis* previously isolated in Austria (GenBank accession number AB461395) and 99.28% to 99.71% homology with *E. multilocularis* previously isolated in France and Slovakia (GenBank accession numbers AB461396 and AB461397, respectively), both of which belong to the European haplogroup. Additionally, these Canadian horses had *cob* sequences close to those of *E. multilocularis* isolated from a Canadian patient with alveolar echinococcosis (GenBank accession number MK843307) and a coyote (GenBank accession number KC550003) that had been reported previously as European-type strains found in Canada (Gesy and Jenkins, 2015; Massolo et al., 2019). When compared with the Austrian isolate (GenBank accession number AB461395), LC764417 had one nucleotide substitution, LC764418 had one nucleotide insertion, LC764419 had two nucleotide substitutions and one nucleotide insertion, and LC764420 had one nucleotide substitution and three nucleotide insertions. When compared with the AB461395 amino acid sequence, these four isolates had 99.47% to 100% homology in the amino acid sequences of the open reading frame region. When compared with AB461395, LC764417, LC764419, and LC764420 have an amino acid mutation in the open reading frame. These four nucleotide sequences and amino acid sequences are shown in Supplementary Table 2.

**Fig. 2:**
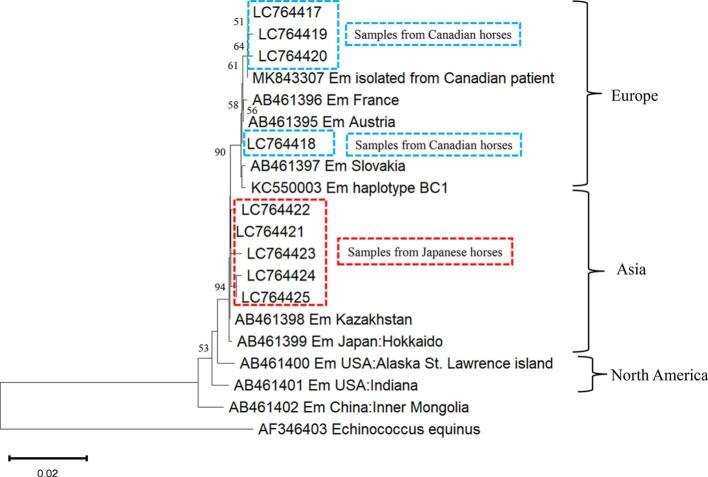
Phylogenetic tree for *E. multilocularis* isolated from horses originating in Japan and Canada based on the partial sequences (694 bp) of *cob* genes. The neighbor-joining method, based on the Tamura-Nei model, was performed. Numbers next to branches indicate the percentage of replicate trees based on 1000 bootstrap replicates. Only bootstrap values ≥50% are shown. The scale bar indicates 0.02 nucleotide substitutions per nucleotide position. The isolates in this study had accession numbers LC764417 to LC764425. The isolates obtained from horses originating in Japan (LC764421 to 764,425) are surrounded by dashed red lines, and those from horses originating in Canada (LC764417 to 764,420) are surrounded by dashed blue lines. In the phylogenetic tree, “Em” is an abbreviation for *Echinococcus multilocularis*.

**Fig. 3:**
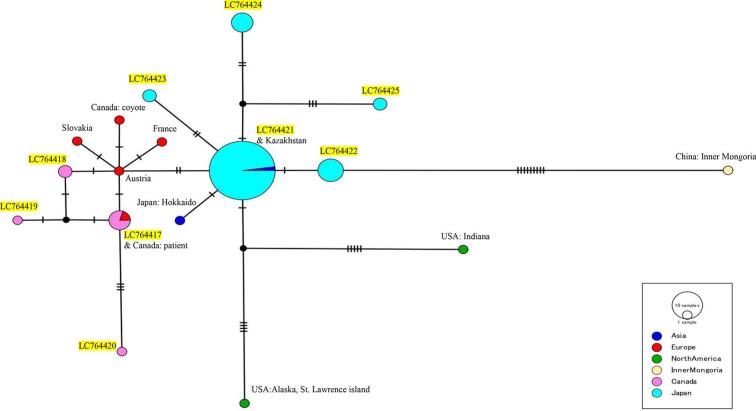
Haplotype network based on the partial sequences (694 bp) of *cob* genes of *E. multilocularis* constructed using PopART version 1.7 software. This analysis shows nine haplotypes, including five haplotypes obtained from horses originating in Japan and four haplotypes obtained from horses originating in Canada. The nine haplotypes obtained in this study are highlighted in yellow. The most common strains isolated from horses originating in Japan and Canada were LC764421 (74.2%) and LC764417 (50.0%), respectively. Each colour represents a unique haplogroup (Asia, North America, Europe, etc.), and the circle size is proportional to the number of isolates contained. One vertical line on the branch indicates a single base mutation. Black circles represent hypothetical haplotypes. The accession numbers of samples used for reference are as follows: AB461398 (Kazakhstan), AB461399 (Japan: Hokkaido), AB461395 (Austria), AB461396 (France), AB461397 (Slovakia), KC550003 (Canada: coyote), MK843307 (Canada: patient), AB461400 (USA: Alaska, St. Lawrence Island), AB461401 (USA: Indiana), AB461402 (China: Inner Mongolia). (For interpretation of the references to colour in this figure legend, the reader is referred to the web version of this article.)

Based on our results, the molecular phylogenetic tree analysis based on the *cob* sequences of *E. multilocularis* showed that *E. multilocularis* isolated from Japanese horses was of the Asian haplogroup, and *E. multilocularis* isolated from Canadian horses was of the European haplogroup (Fig. 2). Fig. 3 shows the results of haplotype network analysis. Among the isolates obtained from the Japanese horses, those corresponding to the Kazakhstan isolate were the most common, comprising forty-six of all sixty-two isolates (74.2%). All other isolates obtained from Japanese horses grouped around the Kazakhstan isolate were separated by one to four single nucleotide variants. On the other hand, among the isolates obtained from the Canadian horses, those similar to the Austrian isolate were most common, containing four of the eight isolates (50.0%). All isolates obtained from Canadian horses grouped around the Austrian isolate were separated by one to four single nucleotide variants.

## 4 Discussion

This study identified the haplogroups of *E. multilocularis* obtained from Japanese and Canadian horses. Alveolar echinococcosis is endemic to both Hokkaido (Eckert and Deplazes, 2004) and Alberta (Catalano et al., 2012; Massolo et al., 2014). According to the previous report (Morishima et al., 1999), in Hokkaido, Japan, the infection rate of *E. multilocularis* in red foxes, which are definitive hosts of *E. multilocularis*, was 53.4%. On the other hand, according to a study conducted in Alberta, Canada where horses were raised prior to being exported to Japan, coyotes, a definitive host for *E. multilocularis*, had an infection rate of 20.5% (Catalano et al., 2012). Based on this information, the difference in the prevalence of alveolar echinococcosis between Japanese (Hokkaido-raised) and Canadian (Alberta-raised) horses in this study may indicate the difference in the contamination status of *E. multilocularis* eggs in the environment where horses are raised.

According to a previous report from Japan, larval *E. multilocularis* infections in horses likely originated in Hokkaido, which is a major production area for horses, and alveolar echinococcosis is endemic (Goto et al., 2010; Hifumi et al., 2015). These results suggest that the slaughtered horses originating in Japan with alveolar echinococcosis in this study had been infected by larval *E. multilocularis* in Hokkaido. The previous report demonstrated that Asian haplotypes of *E. multilocularis* were detected in a fox and a vole in Hokkaido, Japan (Nakao et al., 2009). The *12S rRNA* PCR testing demonstrated that *E. multilocularis* detected in horses originating in Japan showed a high identity to previously reported isolates from Hokkaido, Japan (Goto et al., 2010). Unlike the above-mentioned report, *cob* PCR testing in this study showed higher identity with the Kazakhstan isolate than with the Japan isolate. This suggests that isolates genetically related to the Kazakhstan isolate are widely distributed in Hokkaido, which is a major production area for horses in Japan. Furthermore, the Asian haplotype, which includes *E. multilocularis* reported in Japan and that reported in Kazakhstan, probably originates from St. Lawrence Island, Alaska, USA, according to reports based on molecular phylogenetic analysis of mitochondrial genes of *E. multilocularis* (Alvarez Rojas et al., 2020; Hayashi et al., 2023). Of further note, the isolates we obtained from Japanese horses showed 99.43% to 100% homology with an Asian haplotype (GenBank accession number KY205670) that has also been detected in Poland (Karamon et al., 2017). Such geographical diversity suggests that *E. multilocularis* infection may be spread by the artificial introduction of domesticated animals to different areas in the world (Hayashi et al., 2023).

Conversely, European haplotypes of *E. multilocularis* were isolated from Canadian horses. Based on the previous literature, Canada is one of the countries with the most reported prevalence of alveolar echinococcosis (Schurer et al., 2019). According to one report, the North American, Alaskan, and European haplotypes of *E. multilocularis* were distributed in the North Central region that includes the southern portions of Canada (Alberta, Saskatchewan, and Manitoba) and thirteen contiguous states in the United States (Wu et al., 2017). Over the past decade, the European haplotypes of *E. multilocularis* have been detected in Canada, not only in definitive hosts such as coyotes, but also in humans, raising concerns about the spread of the European haplotype of *E. multilocularis* in Canada (Gesy and Jenkins, 2015; Jenkins et al., 2012; Massolo et al., 2019).

According to Jenkins et al. (2012), the following two possible hypotheses explain the invasion of the European haplotype of *E. multilocularis* in Canada. First, it is possible that dogs imported from Europe carried it. Second, a European haplotype of *E. multilocularis* may have been brought to North America in the last century when red foxes from France and Scandinavia were introduced. It has been reported that the European haplotypes of *E. multilocularis* carry a greater risk of causing human alveolar echinococcosis than do the North American haplotypes mainly distributed in Canada, raising concerns about the further spread of infection to humans (Massolo et al., 2019). Our previous report also demonstrated that the European haplotypes of *E. multilocularis* were isolated from slaughtered horses originating in Alberta, Canada (Hifumi et al., 2021). These findings, and the results of this study, suggest that the definitive hosts of *E. multilocularis* in Alberta, Canada may carry a high proportion of the European haplotypes of *E. multilocularis*. This idea was supported by a recent report (Santa et al., 2023).

The obtained nucleotide sequences (LC764417, LC764419, LC764420, and LC764423) had one or two amino acid mutations in the open reading frame. However, these changes may not influence the protein structure because these mutations do not change the polarity of the amino acids.

## 5 Conclusions

This study suggested that *cob*-gene-targeted PCR testing could be useful for the geographical genetic characterization of *E. multilocularis* isolated from horses. The results obtained in this study through the monitoring of equine alveolar echinococcosis reflect the epidemiological status of *E. multilocularis* in the regions of Japan and Canada where horses are raised for slaughter, and provide useful information from the perspective of public health.

## Declaration of competing interest

The authors declare that they have no known competing financial interests or personal relationships that could appear to have influenced the work reported in this paper.

## Appendix A Supplementary data

Supplementary material 1 S. Fig. 1. Location and sequences of designed primers for PCR testing targeting the *cob* gene of *E. multilocularis.* Supplementary material 1

Supplementary material 2 S. Fig. 2. Representative photograph of histopathology (Case no. K-21). A: The nodule (N) consisted of mature collagen fibers and was well-demarcated from the liver parenchyma (L). Moderate infiltration of inflammatory cells (I) consisting of lymphocytes, eosinophils, and macrophages was observed at the periphery of the nodule. Hematoxylin and eosin (H&E) stain. Bar = 100 μm. B: Several laminated layers (black arrows) were observed at the center of the nodule. H&E stain. Bar = 100 μm. C: Several laminated layers (black arrows) were positive for periodic acid–Schiff (PAS) stain. PAS stain. Bar = 100 μm. Supplementary material 2

Supplementary material 3 S. Fig. 3. Representative photograph of *cob*-gene PCR testing. Cases in which a band was detected at 694 bp were diagnosed as positive. M—size marker; N—negative control (sterile distilled water); P—DNA derived from the protoscolex of *E. multilocularis* used as a positive control. Supplementary material 3

Supplementary material 4 S. Table 1. Data for seventy horses diagnosed with alveolar echinococcosis. Supplementary material 4

Supplementary material 5 S. Table 2. Nucleotide sequences and amino acid sequences obtained from LC764417 to LC764425. AB461398 and AB461395 are used to indicate the Asian haplotype and the European haplotype, respectively. The nucleotide sequences of these references corresponding to an open reading frame are shown in green. The substitutions and insertions of nucleotide sequences and amino acid sequences are shown in red. Supplementary material 5
