# Supplementary material for: Molecular phylogenetic analysis of Echinococcus multilocularis from horses raised in Canada or Japan, using mitochondrial cytochrome b gene–targeted PCR
Source: Food Waterborne Parasitol. 2024 Jan 13;34:e00219. doi: 10.1016/j.fawpar.2024.e00219 (PMC10827676; doi:10.1016/j.fawpar.2024.e00219)
Supplement: Supplementary material 1 — S. Fig. 1. Location and sequences of designed primers for PCR testing targeting the cob gene of E. multilocularis. [file mmc1.docx]

Supplementary Figure1

>AB461399.1 Echinococcus multilocularis mitochondrial cob gene for cytochrome b, complete cds, country: Japan:Hokkaido

ATGATTGTTTTGTTTCGACGTAATTTAATAGATTTACCAATTAATTATTCTTTGAATTATTATTGAAGTA

GTGGGTTTGTATTGTCTATGTTTATGATTCTTCAAATTTTTACTGGAGTATTGTTGTCTTTTTTGTATGT

AGCTGATTTTATGTGTAGATTTTTTATGGTTATGAATTTATCTAATGATTCTTTTTTTACTTGATGTTTG

CGTTATTGGCATATGGTAGGTGTAAATGTACTGTTTATTTTGTTATTCTTTCATATGGGTATGGCTTTGT

ATTATGGTAGTTATGTTAAGAAGGGTGTTTGGAATGTTGGTTTTGTGTTATATTTGTTAGTTATGGGTGA

GGCATTTACTGGTTATATTTTACCTTGGCGTCAGATGTCTTATTGGGCTGCCACTGTCCTTACTTCAATT

GTTGATAGTTTGCCGTTGGTTGGTTCTATGGTCTATAAGTATGTGGTTGGTGGATTTTCGGTGTCGGGTG

TAACATTGATTCGTGTGTTATCGGTTCATATTTGTTTGGGGTTTGTTATATTAGGATTAATGTTTGTTCA

TTTATTTTATCTTCATAAGAGTGGTAACAGTAATCCGTTGTTTTCATTTAATTTGTTTAATGATTTGGTG

TATTTTCATTCATATTTTTCTGTTAAGGATTTAGTCTTGTTTATGTTTACTTGTAGATTGGTAGTTTTTT

GGTTGTTTTTTGCTCCTGATTTATTGGTAGATATAGAGGCATATTTAGAGGCTGATTATCTGAAAACTCC

TGTTAGTATTAAGCCTGAGTGATATTTTTTAGCCTTTTATGCTATTCTTCGGTGTATTAATTCTAAGGTT

GGGGGGTTGTTGTTGATTTTATCTTTTATTTTTTTCTTATGAGTACCAACTGAGGGTGGCACTAGTGTTT

ATAGAGTTTGGCGTCAGGTTAATTTTTGGTTAGTTGTTAGTTTGTTTTTGTCATTGACTTATTTAGGTGG

GTGTCACCCAGAGTATCCTTATTTGGTTATTTGTCAGTTGTTTAGGGTAGTAATGGTGTTTATGATGTTT

GTTTTTAAGTTATATTAA

The positions of sequences used for PCR primers are colored.

PCR forward primer PCR reverse primer
