# Supplementary figures and images for: Molecular phylogenetic analysis of Echinococcus multilocularis from horses raised in Canada or Japan, using mitochondrial cytochrome b gene–targeted PCR
Source: Food Waterborne Parasitol. 2024 Jan 13;34:e00219. doi: 10.1016/j.fawpar.2024.e00219 (PMC10827676; doi:10.1016/j.fawpar.2024.e00219)

## Slide 1
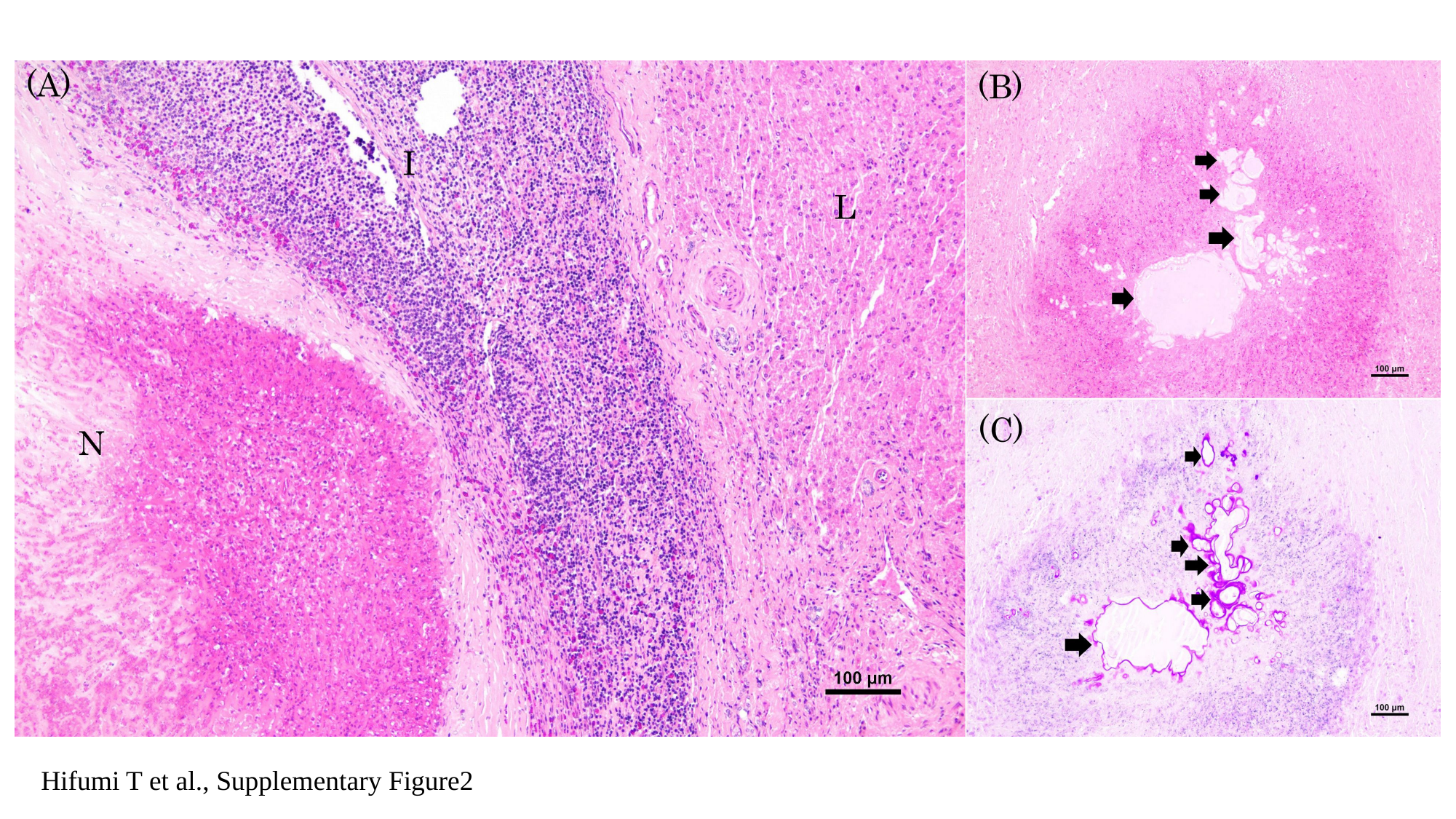

Hifumi T et al., Supplementary Figure2

Supplement: Supplementary material 2 — S. Fig. 2. Representative photograph of histopathology (Case no. K-21). A: The nodule (N) consisted of mature collagen fibers and was well-demarcated from the liver parenchyma (L). Moderate infiltration of inflammatory cells (I) consisting of lymphocytes, eosinophils, and macrophages was observed at the periphery of the nodule. Hematoxylin and eosin (H&E) stain. Bar = 100 μm. B: Several laminated layers (black arrows) were observed at the center of the nodule. H&E stain. Bar = 100 μm. C: Several laminated layers (black arrows) were positive for periodic acid–Schiff (PAS) stain. PAS stain. Bar = 100 μm. [file mmc2.pptx]

## Slide 1
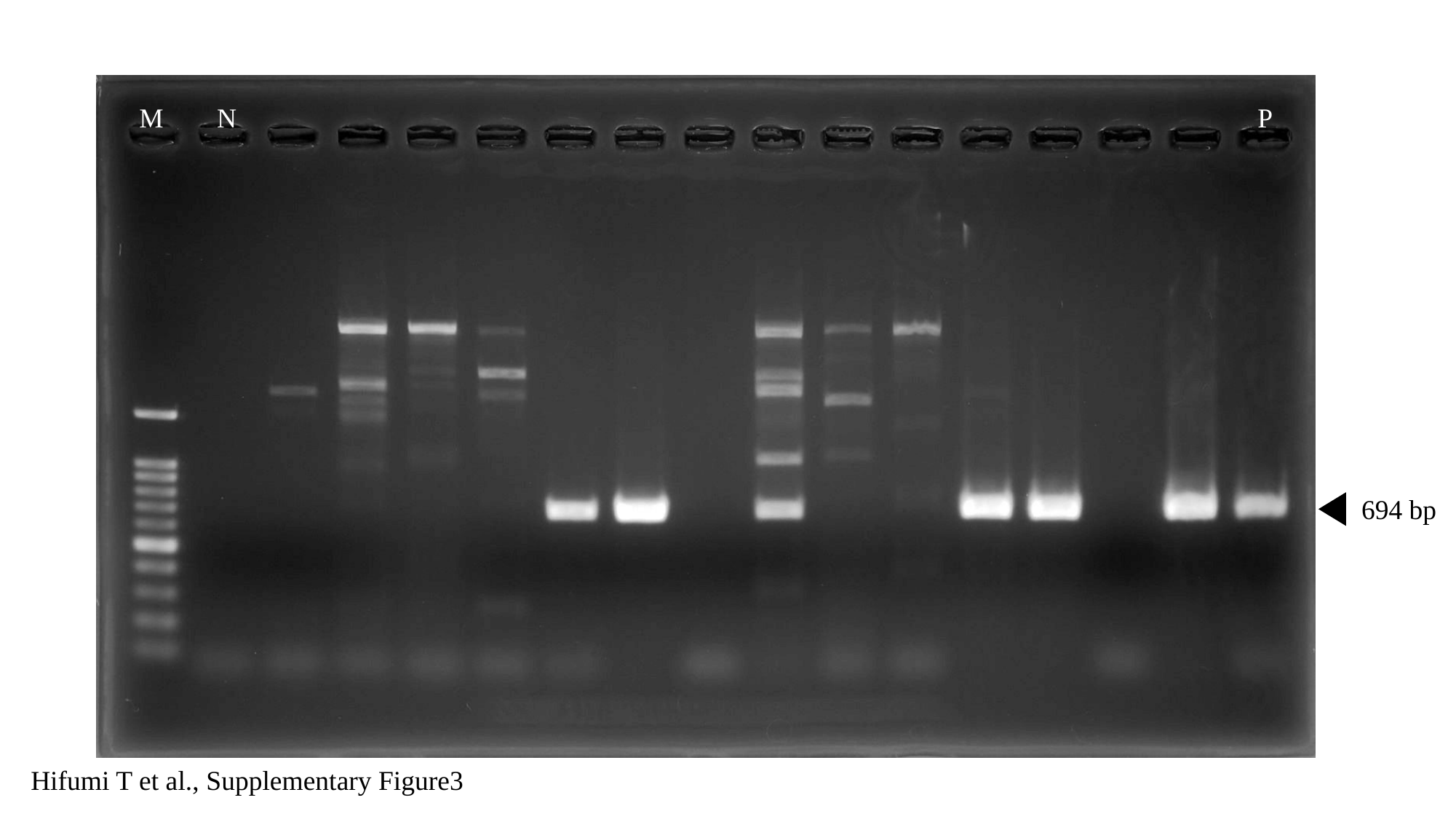

M
N
P
694 bp
Hifumi T et al., Supplementary Figure3

Supplement: Supplementary material 3 — S. Fig. 3. Representative photograph of cob-gene PCR testing. Cases in which a band was detected at 694 bp were diagnosed as positive. M—size marker; N—negative control (sterile distilled water); P—DNA derived from the protoscolex of E. multilocularis used as a positive control. [file mmc3.pptx]
